# Supplementary material for: Insights on Molecular Characteristics of Hydrochars by 13C-NMR and Off-Line TMAH-GC/MS and Assessment of Their Potential Use as Plant Growth Promoters
Source: Molecules. 2021 Feb 15;26(4):1026. doi: 10.3390/molecules26041026 (PMC7919478; doi:10.3390/molecules26041026)
Supplement: Supplementary file 1 [file molecules-26-01026-s001.pdf]

Insights on molecular characteristics of hydrochars by ^{13}C NMR and Off-line TMAH-GC/MS and assessment of their potential use as plant growth promoters

Laís G. Fregolente [a,b], João Vitor dos Santos [a], Giovanni Vinci [b], Alessandro Piccolo [b], Altair B. Moreira [a], Odair P. Ferreira [c], Márcia C. Bisinoti [a], Riccardo Spaccini [b*]

[a] Universidade Estadual Paulista (Unesp), Instituto de Biociências, Letras e Ciências Exatas, Campus de São José do Rio Preto, Laboratório de Estudos em Ciências Ambientais, Rua Cristovão Colombo 2265, Jardim Nazareth, São José do Rio Preto, São Paulo State, Brazil, postcode 15054-000.

[b] Interdepartmental Research Centre on Nuclear Magnetic Resonance (NMR) for the Environment, Agro-food and New Materials (CERMANU), Università degli Studi di Napoli Federico II, Via Università 100, Portici, Italy, postcode 80055.

[c] Universidade Federal do Ceará, Laboratório de Materiais Funcionais Avançados (LaMFA), Fortaleza, Ceará State, Brazil, P.O. Box 3151, postcode 60455-900.

Figure S1. Effect of hydrochar soluble fraction concentration (C_0 = 0 mg C L^{-1}, C_1 = 1 mg C L^{-1}, C_{10} = 10 mg C L^{-1}, C_{50} = 50 mg C L^{-1}, C_{100} = 100 mg C L^{-1}) on germination percentage of maize seeds during 7 days.

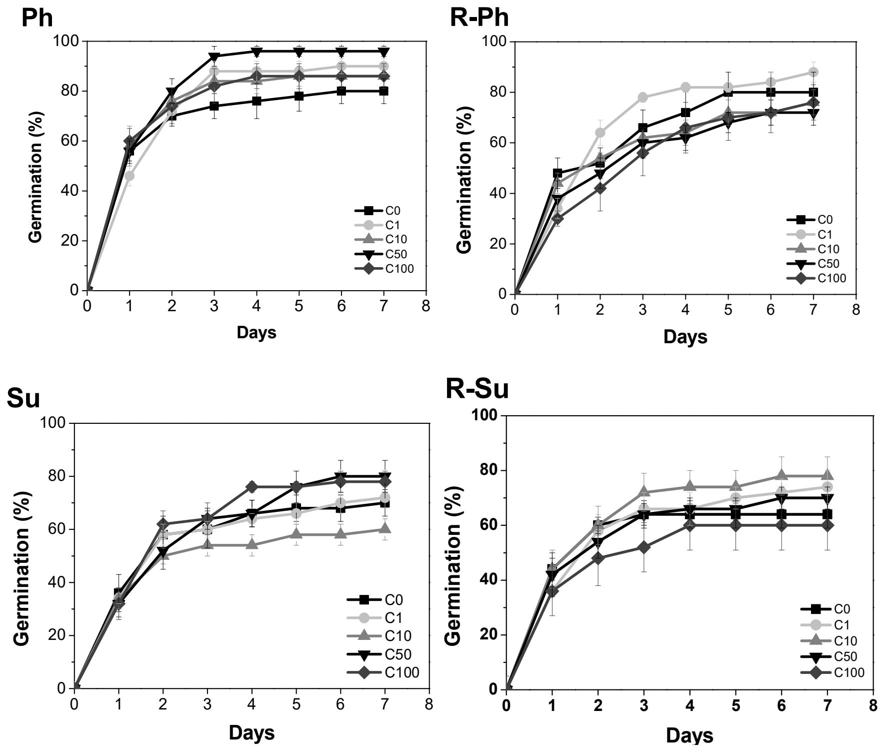

Table S1. Compounds released by thermochemolysis- GC/MS of hydrochars, with a relative area percentage ≥ 0.50%, the retention time (RT) recorded and the degree of matching/probability (P) in percentage for each compound.

		**HYDROCHAR**							
**RT**	**COMPOUND**	**Ph**		**R-Ph**		**Su**		**R-Su**	
			**P (%)**		**P (%)**		**P (%)**		**P (%)**
5.34	Benzene,1-methoxy-4-methyl	X	91.9	X	91.2	X	78.9		
5.36	2-butenedioic acid (E)-,dimethyl ester							X	85.1
5.48	butanedioic acid, dimethyl ester	X	93.5	X	94.7	X	93.1	X	86.3
5.81	2',6'-dihydroxy-3'-methylacetophenone					X	80.2	X	79.2
5.97	phenol 2-methyl	X	93.5	X	91.2	X	90.2		
6.06	butanedioic acid, methyl-,dimehtyl ester	X	81.9	X	83.0	X	80.5		
6.19	Benzenamine,2-methoxy-	X	88.1	X	88.0				
6.37	phenol,4-methyl-	X	92.4	X	91.7	X	91.9		
6.40	phenol, 3-propyl-					X	82.8		
6.54	mequinol	X	90.3	X	89.8				
6.64	butanedioic acid, methylene-, dimethyl ester					X	82.6		
6.72	benzoic acid, methyl ester			X	82.7	X	69.7	X	81.7
6.95	methyl-2-thiophene carboxylate							X	86.8
6.96	dimethyl ethylidenemalonate					X	75.2		
7.06	2-thiophenecarboxylic acid hydrrazide							X	82.5
7.07	benzene,1-ethyl-4-methoxy	X	93.5	X	94.7	X	93.4		
7.47	pentanedioic acid, dimehtyl ester	X	78.8	X	78.7			X	75.5
7.68	benzene, 1,2-dimethoxy-	X	85.7	X	86.0				
7.80	silane, triethyl(4-methylphenoxy)-							X	85.2
7.88	phenol,2,4-dimethyl-					X	88.7	X	89.1
7.94	benzene,1-ethenyl-4-CH3O	X	92.5	X	93.6	X	92.4		
8.15	benzene, 1,4-dimethoxy-	X	89.5	X	87.2				
8.47	phenol,2-methoxy-3-methyl			X	76.4				
8.80	phenol,4-methoxy-3-methyl			X	79.0				
9.16	benzenepropanoic acid,2-methoxy-,methyl ester					X	70.0		
9.22	benzoic acid,3-methyl-,methyl ester							X	89.8
9.37	2-thiophenecarboxylic acid, 5-methyl-,methyl ester							X	90.3
9.68	2-thiophenecarboxylic acid, 5-methyl-,methyl ester							X	86.6
9.89	2,6-dimethoxytoluene	X	92.5						
10.15	phenol,2-methyl-6-(2-propenyl)-			X	80.2				
10.54	benzaldehyde,4-methoxy-			X	81.4			X	87.9
10.87	benzene,1-methoxy-4-(1-methylpropyl)-							X	77.7
10.94	2,6-dimethoxytoluene			X	71.7				
11.18	benzoic acid,2,3-dimethyl-,methyl ester							X	92.3
11.79	1,2,3-trimethoxybenzene	X	93.9	X	94.1				
12.10	2,5-dimethoxyethylbenzene	X	89.5	X	90.1				
12.36	benzene,1-methoxy-4-(methylthio)-							X	89.6
12.60	benzoic acid,4-methoxy-,methyl ester	X	90.7						
12.64	benzoic acid,3-methoxy-,methyl ester			X	94.3	X	93.5	X	92.7

12.91	phenol,2,6-dimethoxy	X	90.2	X	86.3				
13.10	acetophenone,4'-methoxy-	X	91.5	X	91.6				
13.37	1,2,4-trimethoxybenzene	X	89.7	X	94.1				
13.48	Benzene-4-ethenyl-1,2-dimethoxy-	X	84.6	X	92.5				
13.50	4-(para-totyl)-butyric acid							X	85.6
13.66	benzoic acid,4-methoxy-,methyl ester	X	93.4	X	93.9				
14.29	benzene,1,2,3-trimethoxy-5-methyl	X	91.5	X	92.9				
14.64	trimethyl 1,2,3-propanetricarboxylate	X	88.3	X	86.6	X	90.1	X	89.9
15.18	benzoic acid, 2-methoxy-, methyl ester							X	80.6
15.63	1,3-benzodioxole-5-carboxylic acid, methyl ester							X	75.1
15.72	dimethyl phthalate			X	73.8				
16.12	3-(2-methoxy-5-methylphenyl)propanoic acid	X	82.0	X	80.9				
16.61	2,3-thiophenedicarboxylic acid, dimehtyl ester							X	86.3
17.08	benzenebutanoic acid,2,5-dimethyl-							X	81.6
17.18	1,4-benzenedicarboxylic acid,dimethyl ester							X	91.3
17.48	cis-2-(3,4-Di-CH3O phenyl)-1-CH3O ethylene	X	-	X	-				
17.60	dimethyl 2,5-thiophenedicarboxylate							X	85.3
17.85	C12 FAME					X	80.1	X	69.2
17.97	benzenepropanoic acid,4-methoxy-,methyl ester	X	92.5	X	93.4				
18.48	C11-OCH3			X	-				
18.52	nonanedioic acid, dimethyl ester							X	87.6
18.98	ethanone,1-(3,4-dimethoxyphenyl)-	X	83.8	X	82.7				
19.72	benzoic acid, 3,4-dimethoxy-, methyl ester	X	94.6	X	94.6			X	84.8
20.60	1,2-benzenedicarboxylic acid,4-methyl- ,dimethyl ester							X	83.3
21.30	sebacic acid monomethyl ester							X	86.0
22.02	2-propenoic acid,3-(4-methoxyphenyl)-,methyl ester, (E)-	X	86.9	X	88.4				
23.14	3,4,5-Tri-CH3O benzoic acid ME	X	70.0	X	97.5				
23.38	C14 FAME					X	84.0		
25.26	C15 anteiso FAME							X	-
25.41	C18 alkane			X	64.7				
26.00	C15 FAME					X	86.6		
28.52	C16 FAME			X	82.8	X	82.2	X	71.5
30.66	5-methoxy-2-methyl-4-oxo-1,2,3,4-tetrahydro-1,10,phenantthrolin			X	74.6				
30.94	C17 FAME					X	80.8		
32.78	C18:1 FAME					X			
33.24	C18 FAME	X	76.1	X	74.5	X	80.1	X	75.2
33.50	nonanoic acid, 9-(O-propylphenyl)-,methyl ester					X	74.9		
36.00	hexadecanedioic acid, dimethyl ester							X	69.6
37.25	octadecanoic acid, 4-hydroxy-,methyl ester					X	82.1		
37.59	C20 FAME	X	80.4	X	80.4	X	82.8		
41.57	1,2-benzenedicarboxylic acid, mono(2-ethylehyl) ester	X	85.9						
42.97	C26 alkane					X	65.8		
43.52	tricosanoic acid, methyl ester					X	85.9	X	83.1
44.48	esterol					X	60.8		

44.80	C27 Alkane			X	-	X	-	X	-
45.34	C24 FAME	X	83.2	X	76.7	X	79.7	X	79.3
45.45	1,3-benzenedicarboxylic acid,bis(2-ethylhenyl) ester			X	85.3	X	83.5	X	87.3
46.51	C26:1 FAME	X	-	X	-	X	-		
46.59	C28 alkane	X	-	X	77.9				
47.10	C26-OCH3	X				X	-	X	-
47.36	C24, 2-CH3O, FAME					X	-		
48.31	C28 alkane							X	79.6
48.82	C26 FAME	X	90.2			X	90.2	X	79.3
49.95	C30 alkane			X	81.0				
49.31	C24,24-CH3O, FAME					X	-		
50.49	C28-CH3O	X	-	X	-			X	-
51.58	Alkane					X	-		
52.12	C28 FAME	X	85.7	X	85.7	X	86.5	X	86.4
53.13	C32 alkane			X	79.4				
53.62	C30-CH3O	X	-			X	-	X	-
55.18	C30 FAME	X	84.3	X	83.8	X	83.9	X	70.2

FAME=fatty acid methyl ester; ME= Methyl ester; CH3O = methoxy, (-) The attribution was made based in the main peaks identified.

Table S2. Germination index of maize seeds calculated for each concentration of hydrochar soluble fraction for Ph, R-Ph, Su and R-Su samples.

Germination Index (GI)

Ph		R-Ph		Su		R-Su	
**r= 0.83**		**r= -0.97**		**r= 0.27**		**r= 0.43**	
C0	6.5±0.4	C0	5.8±0.6	C0	5.0±0.6	C0	5.3±0.4
C1	6.5±0.2	C1	5.6±0.3	C1	4.9±0.1	C1	5.1±0.3
C10	7.0±0.6	C10	5.4±0.3	C10	4.4±0.2	C10	5.7±0.6
C50	7.4±0.3	C50	4.9±0.6	C50	5.1±0.5	C50	5.4±0.5
C100	7.2±0.5	C100	4.5±0.5	C100	5.1±0.4	C100	4.5±0.7

*r=correlation coefficient
